# Ophthalmomyiasis Case Caused by Two Blow Fly (Diptera: Calliphoridae) Species in North America

**DOI:** 10.1155/2024/2209301

**Published:** 2024-05-14

**Authors:** Taylor B. Parker, Kelly A. Meiklejohn, Gregory A. Dahlem, Ralph C. Eagle, Marius J. Heersink

**Affiliations:** ^1^Department of Population Health and Pathobiology, North Carolina State University, 1060 William Moore Drive, Raleigh, NC 27606, USA; ^2^Department of Biological Sciences, Northern Kentucky University, Nunn Drive, Highland Heights, KY 41099, USA; ^3^Department of Pathology, Wills Eye Hospital, 840 Walnut Street, Philadelphia, PA 19107, USA; ^4^Department of Ophthalmology, Wills Eye Hospital, Sidney Kimmel Medical College of Thomas Jefferson University, 840 Walnut Street, Philadelphia, PA 19107, USA

## Abstract

Ophthalmomyiasis is the result of fly larvae feeding on the tissues of the eye. Commonly associated with poor hygiene and open wounds, this condition is rare and often stigmatized. Treatment can be straightforward, and full recovery is common. Identifying the species responsible for ophthalmomyiasis is important for the medical, forensic, and entomological communities. Here, we present a case of ophthalmomyiasis where 30–40 blow fly (Diptera: Calliphoridae) larvae were removed from the eye of a human male. A representative subsample of five larvae was used for taxonomic identification via two approaches (a) DNA analysis, via sequencing of the complete mitochondrial genome (mtGenome) and comparison of the mtGenome and mitochondrial *COI* barcode region to GenBank, and (b) morphology, examination of the posterior spiracles using microscopy, and comparison to published larval descriptions of blow flies. Two species of blow flies were identified from the DNA analysis: *Lucilia coeruleiviridis* and *Phormia regina*. Morphological examination could only confirm *L. coeruleiviridis* as being present. To our knowledge, finding two blow fly species causing ophthalmomyiasis in a single individual has not been previously reported in the scientific literature. Neither *P. regina* nor *L. coeruleiviridis* prefers living tissue for larva development, but since they fill similar ecological niches, perhaps this was a show of competition rather than a normal feeding habit. Knowing these blow fly species can resort to this behavior, and that it can affect human populations, is valuable to the education of patients and providers.

## 1. Introduction

Myiasis is a type of parasitism that results from dipterous larvae developing in live vertebrate tissue. Larvae across many species within the fly family Calliphoridae—commonly known as blow flies—are known for having diverse feeding habits, ranging from necrophages feeding on decayed matter (including human bodies), to obligate parasites feeding exclusively on live tissue, and facultative parasites which can feed on live or dead animal matter (depending on environmental conditions). For species reported to cause obligate parasitism, adult flies will commonly lay their eggs in moist areas of tissue, such as wounds or entrances to the body, and the larvae will feed on the tissue to continue through development.

Ophthalmomyiasis is an uncommon and underdocumented phenomenon due to surrounding stigmas. This condition specifically refers to the infestation of (peri-)ocular tissue by fly larvae. This is most commonly known to occur with the larvae of the human botfly (*Dermatobia hominis*) (e.g., [1, 2]) and the sheep nasal botfly (*Oestrus ovis*) (e.g., [3, 4]) but has been documented in other fly genera (e.g., *Lucilia* (Calliphoridae) and *Sarcophaga* (Sarcophagidae)) [4]. Most patients present with ophthalmomyiasis externa, referring to larvae infestation of the external structures of the eye, including the cornea and conjunctiva. Some patients may progress to ophthalmomyiasis interna, when larvae penetrate the eye, although this is rare [5]. The condition is most commonly observed in (sub)tropical regions and is associated with poor hygiene and close animal contact. Other common risk factors for ophthalmomyiasis include ocular wounds, advanced age, debilitation, and poor access to nutrition and care. Given the rarity and health impacts of ophthalmomyiasis, when cases are encountered in practice, it is important to identify the species present.

Morphological identification is the gold standard method of taxonomic identification of a range of organisms including insects. Although reliable and low-cost, there are two major hurdles to the routine identification of blow fly larvae using morphology: (a) there are a limited number of experts globally and (b) the majority of species lack distinguishing characteristics visible using a standard light microscope. Given this, it can be difficult to reliably identify blow fly larvae, especially at the species level. DNA analysis can be used as a supplemental or alternative method for taxonomic identification. Typically, regions from the mitochondrial genome (mtGenome) are targeted to permit DNA-based species identifications in insects, including blow flies, given (a) the high copy number of the mtGenome means recovery of mitochondrial DNA (mtDNA) is likely even in highly compromised samples, (b) the mtGenome shows accelerated rates of evolution which can be harnessed to permit discrimination even among closely related taxa, and (c) there are sufficient mitochondrial sequences available in public databases (e.g., GenBank [6] and BOLD [7]) to allow comparisons of unknowns [8].

In this paper, we document a case of ophthalmomyiasis and outline the DNA-based and morphological approaches implemented to identify the blow fly species present.

## 2. Materials and Methods

### 2.1. Case Report

A 59-year-old Caucasian male was referred to a tertiary-care center in Philadelphia, PA, for right eye pain and redness with mild blurry vision. The patient complained of “bugs crawling on my face” and eye redness for approximately one week. Of note, the patient had previously been treated for clinical alcoholism and was unhoused at the time of presentation.

**Figure 1:**
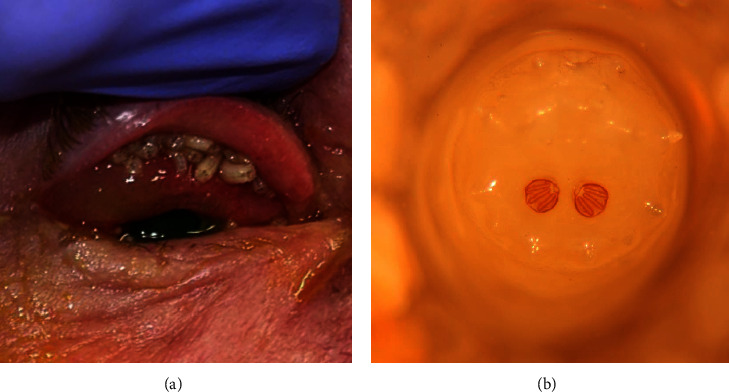
Photograph of blow fly larvae present inside the patient's upper fornix (a). Light microscopy image depicts the posterior spiracles belonging to one of the *Lucilia coeruleiviridis* specimens removed from the eye and used for morphological taxonomic assignment (b).

On first presentation, multiple white larvae were noted to crawl out of the patient's right eye when the eye was manipulated. The patient's vision was measured to be 20/30 in the right eye and 20/20 in the left eye by Snellen acuity. External examination showed 1-2+ injection and chemosis. On upper eyelid eversion, approximately 30 to 40 larvae were noted to emerge from the patient's upper fornix (Figure 1(a)). Lower forniceal exam was normal. With fluorescein staining, most of the superior conjunctiva and approximately 80% of the cornea were found to have an epithelial defect without corneal stromal infiltrate or thinning. Pupil examination, extraocular motility, intraocular pressure, and anterior segment examination were normal. Dilated fundoscopic examination was normal, without evidence of intraocular infestation by larvae.

The patient was then given topical anesthesia, and all visible larvae were removed bedside with blunt tip forceps. Approximately 40 living larvae were collected in specimen cups. Upper and lower fornices were swept with a cotton-tip applicator multiple times to ensure no larvae remained. The patient was then treated with aggressive applications of antibiotic ointment every 3 hours to promote reepithelialization and to suffocate any remaining larvae. The patient remained inpatient for 3 days, and each day was examined with improving clinical exam and without evidence of further larvae infection. On the last day of admission before signing out of the hospital against medical advice, the patient's epithelium had completely healed, conjunctival injection and chemosis had resolved, and the patient's vision returned to 20/20 by Snellen acuity. The patient did not return for outpatient follow-up examination.

### 2.2. Identification of Larvae

#### 2.2.1. DNA Analysis

Five larvae were selected for DNA analysis that represented diversity with regard to size and developmental stage (e.g., second- or third-instar; Table 1). Using a sterile scalpel, the anterior third of each larva was dissected and used as an input for DNA isolation using the DNeasy Blood and Tissue Kit (Qiagen, Hilden, Germany) according to the manufacturer's instructions [9]. The quantity of total genomic DNA recovered from each larva was determined using the Qubit 3 Fluorometer (Invitrogen, Waltham, MA, USA) and the Qubit dsDNA kit (Invitrogen). The mtGenome of each sample was sequenced to permit taxonomic identification and briefly involved the following steps: (1) DNA libraries were constructed from isolated total genomic DNA using half reactions of the KAPA Hyper Prep Kit (Roche, Basel, Switzerland) and KAPA Illumina compatible unique dual-indexed adapter kit (Roche) following the manufacturer's recommendations [10, 11], (2) mtDNA was enriched using a custom hybridization capture panel designed off *Calliphora vicina* (Calliphoridae; GenBank accession NC_019639.1; Daicel Arbor Biosciences, Ann Arbor, MI, USA) following the “High Sensitivity” protocol in the Hybridization Capture for Targeted NGS manual [12] without modification, (3) libraries enriched for the mtGenome were sequenced on an Illumina MiniSeq (Illumina, San Diego, CA, USA) using a Mid Output kit (2 X 150 paired end sequencing), and (4) raw sequence reads were imported into the CLC Genomics Workbench (Qiagen) and the mtGenome for each sample was assembled using a modified pipeline from Molto et al. [13] using reference mtGenomes from various blow fly species. Published primers [14] were used to locate the cytochrome oxidase subunit I (*COI*) barcode region from each mtGenome alignment, given this region is often used for species identification in insects. Both the mtGenome and *COI* barcode regions from each sample were individually searched against GenBank using the default parameters of the Standard Nucleotide Basic Local Alignment Search Tool (*blastn*). To interpret the *blastn* results and determine the appropriate level of taxonomic assignment, the Organization of Scientific Area Committees proposed standard *2021-S-0006 Standard for the Use of GenBank for Taxonomic Assignment of Wildlife* [15] was consulted. The remaining posterior two-thirds of each larva were subjected to morphological examination for species identification.

#### 2.2.2. Morphology

The larval remains were identified using published figures of third instar larvae of Calliphoridae [16], a pictorial key to fly larvae of public health importance [17], and personal notes from blow fly rearings by G. Dahlem. Only the posterior portion of the larvae was available for morphological study, as the anterior portion was destroyed for the DNA analysis. This meant that structures such as the cephalopharyngeal skeleton and the prothoracic spiracles were not available for examination. Features of the posterior spiracular pit and posterior spiracles were available, and taxonomic identifications were based on these larval features. Morphological identification was completed in a blinded fashion; details of DNA analysis were not provided to prevent biasing of the morphological results.

## 3. Results

For each sample, a complete mtGenome (excluding the variable control region) was recovered totaling >14,900 base pairs in length (Table 1). When utilizing the mtGenomes to search GenBank, three larvae were taxonomically identified at the species level as *Lucilia coeruleiviridis* (Calliphoridae; ID # 2, 4, 5; Table 1) and two as *Phormia regina* (Calliphoridae; ID # 1 and 3; Table 1). The percentage of identical bases for each mtGenome generated in this study when compared to the sequences contained within GenBank was ≥99%. These assignments were further verified by phylogenetic placement, whereby the mtGenomes from the five larvae specimens in this study clustered with reference mtGenomes from *L*. *coeruleiviridis* and *P*. *regina* downloaded from GenBank (results not shown). When utilizing only the 658-bp *COI* barcode region for taxonomic assignment, sample ID #1 and 3 were also assigned to the species level based on the *blastn* results as *P. regina* (Table 1). However, genus-level assignment to *Lucilia* was only possible for larvae ID # 2, 4, and 5; there are multiple *Lucilia* species whose *COI* barcode region sequences are 100% identical, including *L. coeruleiviridis*, *L. mexicana*, and *L. eximia*, making species level assignment using only this region not possible.

Morphological identification was completed on the posterior two-thirds of each of the five larvae subjected to DNA analysis (Table 1). The specimen representing sample ID #1 was a second instar larva, and no figures were available to identify that specimen to the species level; thus, no taxonomic identification was rendered for this sample (Table 1). The remaining samples were all third instar larvae, which could be compared to published illustrations. Morphologically, samples ID #2, 4, and 5 (Table 1) corresponded to *L. coeruleiviridis* using the structure of the posterior spiracles (Figure 1(b)). Sample ID #3 (Table 1) was initially identified as *L. coeruleiviridis* because it showed a complete peritreme. Published figures of *P. regina* posterior spiracles indicate an incomplete peritreme, not enclosing the button. The specimen of sample ID #3 was more lightly sclerotized, which may indicate that it recently molted to the third instar and may account for the discrepancy with respect to the peritreme. A published photo of a third instar *P. regina* larva [18] seems to show a complete peritreme, which is different from what has been illustrated in other publications. Considering this variability, we could not confidently identify sample ID #3 to the species level, and thus, it was only classified as Calliphoridae (Table 1).

## 4. Discussion

Healthy individuals who have average hygiene are not likely to experience myiasis. Ophthalmomyiasis is an uncommon form of myiasis and, to our knowledge, finding two blow fly (Calliphoridae) species in the same individual has not been previously reported in the scientific literature. Moreover, *Phormia* and *Lucilia* species are from separate Calliphoridae subfamilies, Chrysomyinae and Luciliinae, respectively. Neither *P. regina* nor *Lucilia* sp. prefers living tissue over decaying tissue for larval growth development [19]. However, *P. regina* and *L. coeruleiviridis* are competitors, reported to fill the same niche in the same environment [20].

Our results highlighted that the *COI* barcode region did not permit species-level resolution among closely related *Lucilia* species. Given it has been previously reported that there is not enough genetic variability to distinguish between *L. coeruleiviridis* and *L. mexicana* using only the *COI* barcode region [21], reduced taxonomic resolution with this region was not unexpected in this study. Notably, *L. coeruleiviridis* is most commonly found in the eastern part of the United States (where the patient in this study resided), whereas closely related *L. mexicana* and *L. eximia* are mostly found in the western United States and Central/South America, respectively [22–24]. These observations suggest, in concordance with our mtGenome results, that the three *Lucilia* larvae are most likely *L. coeruleiviridis.*

The morphological identification of the blow fly larvae, which is difficult for even an experienced entomologist, gave consistent results for the three samples we found belonging to the *Lucilia* genus. However, the two samples identified as *P. regina* using DNA analysis could not be confirmed using morphological identification as they were especially small, one being a second instar and the other may have recently molted to third instar. In future ophthalmomyiasis cases, it would be beneficial to perform the morphological identification before destructive DNA analysis. This would ensure that all informative features of the larva useful for taxonomic assignment could be examined. Despite this, we concluded that it was beneficial to present the results of the morphological identification of the posterior two-thirds of each of the five larvae to support our DNA analysis results, due to the rarity of finding two species in the same case.

## 5. Conclusion

This is the first reported instance in which two different genera of blow flies were found in the same human ophthalmomyiasis case. Using mtGenome sequences, we were able to taxonomically assign the larvae to either *P*. *regina* or *L*. *coeruleiviridis;* these results were largely confirmed via *COI* analysis and morphology. Ocular myiasis is rare but preventable and treatable. Education of both patients and providers around the world is necessary to decrease the number of myiasis cases. Identifying the myiasis agent furthers forensic and entomological research, as well as informs medical professionals on species that could cause harm to patients.

## Figures and Tables

**Table 1:** Taxonomic assignment of unknown larvae using DNA-based (whole mitochondrial genome and *COI* barcode region) and morphological approaches.

Sample #	Larval attributes		Taxonomic assignment									
	Instar	Specimen length (mm)	Whole mitochondrial genome					*COI* barcode region				Morphology^*∗*^
			Sequence length (bp)	Query coverage (%)	Identity (%)	*E*-value	Taxonomic assignment	Query coverage (%)	Identity (%)	*E*-value	Taxonomic assignment	Taxonomic assignment
1	2nd	4	14,906	100	99.93	0.0	*Phormia regina*	100	100	0.0	*Phormia regina*	Unknown
2	3rd	4	14,893	100	99.97	0.0	*Lucilia coeruleiviridis*	100	100	0.0	*Lucilia* sp.	*Lucilia coeruleiviridis*
3	3rd	2	14,906	100	99.92	0.0	*Phormia regina*	100	100	0.0	*Phormia regina*	Unknown^
4	3rd	6	14,893	100	99.99	0.0	*Lucilia coeruleiviridis*	100	100	0.0	*Lucilia* sp.	*Lucilia coeruleiviridis*
5	3rd	5	14,893	100	99.97	0.0	*Lucilia coeruleiviridis*	100	100	0.0	*Lucilia* sp.	*Lucilia coeruleiviridis*

Match statistics from GenBank *blastn* searches are given. bp, base pair; *COI*, cytochrome oxidase subunit I. ^*∗*^based on posterior two-thirds of the specimen. ^only taxonomically assigned to the family level (Calliphoridae).

## Data Availability

Mitochondrial genome sequences generated in this study will be made available upon request to the corresponding author.
